# Transcriptomic and Weighted Gene Co-Expression Network Analysis Reveals Molecular Regulatory Mechanisms of Cold Stress in Rice

**DOI:** 10.3390/genes17060639

**Published:** 2026-05-31

**Authors:** Bo Ma, Haoqiang Du, Kefei Tan, Jifang Hu, Xingyu Wang, Kai Liu, Rui Liu, Dongxue Mi, Yixuan Ma, Yongcai Lai

**Affiliations:** 1 Qiqihar Branch of Heilongjiang Academy of Agricultural Sciences, Qiqihar 161006, China; 2 Northeast Branch of National Saline-Alkali-Tolerant Rice Technology Innovation Center, Harbin 150086, China; 3 School of Economics and Management, Qiqihar University, Qiqihar 161006, China; 4 Qiqihar Institute of Engineering, Qiqihar 161006, China; 5 College of Life Sciences, Agriculture and Forestry, Qiqihar University, Qiqihar 161006, China

**Keywords:** rice, cold stress, ROS, WGCNA, transcriptome

## Abstract

**Background**: Cold stress is a major abiotic factor limiting rice growth and yield. Elucidating the molecular mechanisms underlying cold tolerance is therefore of great significance for variety improvement. This study focused on the cold-tolerant variety QJ10 and the cold-sensitive variety DHX2, systematically comparing their physiological and transcriptomic differences under cold stress and identifying genes and modules associated with the cold stress response. **Methods**: After 0, 3, 5, and 7 days of treatment at 4 °C, we measured leaf MDA and Pro contents, as well as SOD and POD activities. We performed multi-time-point transcriptome sequencing using RNA-seq, and conducted data mining and validation through differential expression analysis, Mfuzz trend clustering, WGCNA co-expression network analysis, GENIE3 regulatory network prediction, and qRT-PCR. **Results**: Compared with DHX2, QJ10 exhibited lower MDA levels and higher levels of Pro, SOD, and POD under cold stress. Transcriptome analysis identified a total of 13,599 differentially expressed genes. Trend clustering revealed that QJ10 primarily maintained genes associated with growth, development, and basal metabolism, whereas DHX2 tended to activate defense responses. WGCNA identified the MEturquoise module, which showed significant positive correlations with both cold treatment duration and the activities of SOD and POD. Genes in this module were significantly enriched in pathways such as carbon metabolism, photosynthesis, and ion transport. Twelve key transcription factors were identified, nine of which were highly expressed at the late stage of cold stress in QJ10. GENIE3 further predicted seven key regulatory factors centered on *OsNAC2*, *OsLBD*, and *OsARF19*; the expression patterns of these factors were validated by qRT-PCR and were consistent with the transcriptomic results. **Conclusions**: This study revealed that the cold tolerance of QJ10 is associated with enhanced antioxidant capacity, upregulation of genes related to carbon metabolism, and the induced expression of specific transcription factors. The key transcription factors identified here provide candidate genes for studying the molecular mechanisms of cold tolerance in rice. However, their regulatory functions require further experimental validation.

## 1. Introduction

In nature, plants have evolved a suite of complex physiological and molecular mechanisms to withstand environmental fluctuations. Among abiotic stresses, cold stress critically constrains plant growth, development, and geographical distribution [1]. Cold stress disrupts cellular homeostasis, causing membrane damage, oxidative stress, and metabolic imbalance, thereby impairing normal plant growth and development [2]. Upon cold stress, intracellular levels of reactive oxygen species (ROS) rise markedly, causing cellular damage [3]. To counteract this abiotic stress, plants have evolved a complex regulatory system for ROS homeostasis, in which superoxide dismutase (SOD) and peroxidase (POD) serve as integral components [4]. As a warm-season crop, rice is highly susceptible to low temperatures. In temperate growing regions, chilling stress has become a major yield-limiting factor, and in severe cases, can reduce yields by up to 38.6% [5]. Furthermore, diurnal temperature fluctuations between cold nights and warm days amplify cold-induced injury, reducing rice yields by up to 82% [6]. This pronounced cold sensitivity arises largely from the absence of efficient cold acclimation mechanisms in subtropical and tropical plants, which confers extremely limited tolerance to cold stress [7]. In semi-arid regions with pronounced diurnal temperature fluctuations, abrupt thermal shifts can further exacerbate physiological injury in plants [8]. Studies have shown that under heat stress, wheat (*Triticum aestivum* L.) seedlings exhibit significant reductions in plant height and yield, by 18.45% and 20.12%, respectively [9]. Against the backdrop of global warming, the increasing frequency of extreme cold events has made cold stress a key environmental constraint on rice growth, development, and yield stability.

Transcription factors are pivotal regulators of plant abiotic stress responses, with the *ICE*-*CBF*-*COR* cascade constituting the central pathway for cold stress signaling [10]. ICE1, a bHLH family transcription factor, binds to the *MYC* cis-element in *CBF* promoters, thereby activating downstream *CBF* expression [11]. *CBFs* (C-repeat binding factors), comprising *CBF1*/*DREB1B*, *CBF2*/*DREB1C*, and *CBF3*/*DREB1A*, are core members of the *AP2*/*ERF* transcription factor superfamily. Upon cold stress, these factors specifically recognize and bind *CRT*/*DRE* cis-elements in the promoters of downstream *COR* genes, thereby activating their expression [12]. *CORs* (cold-responsive genes) encode a diverse array of effector proteins, including cold-responsive chloroplast proteins, late embryogenesis abundant *(LEA*) proteins, cold-induced proteins (*KIN*), and osmoregulatory factors. These proteins collectively enhance cold tolerance by preserving membrane integrity, maintaining osmotic homeostasis, and scavenging reactive oxygen species [13]. Hormones also serve as crucial regulators of plant cold stress responses. Upon cold exposure, plants can bolster their tolerance by modulating the biosynthesis and signaling of hormones such as ABA and SA [1,14]. The convergence of multiple pathways highlights the complexity of cold-tolerance mechanisms, enabling plants to reconcile growth with stress resistance.

Despite the identification of numerous cold-stress-associated genes through high-throughput sequencing and population genetic approaches such as GWAS, precisely mapping the specific genes that govern complex traits like cold tolerance remains a formidable challenge [15,16,17]. Weighted gene co-expression network analysis (WGCNA) can offer deeper insight into the co-expression modules and key regulatory genes underlying plant responses to abiotic stress [18]. Wang [19] et al. compared the transcriptional profiles of cold-tolerant and cold-sensitive rice varieties under cold stress using RNA-Seq and WGCNA, revealing the cold tolerance mechanism in japonica rice at the booting stage and identifying key responsive genes. Transcriptomic analysis revealed that genes and transcription factors (*MYB73*, *ERF017*, *MYB30*, and *OBP1*) associated with hormone metabolic pathways—including jasmonic acid, ethylene, abscisic acid, and salicylic acid—coordinately regulate the cold stress response in cotton [20]. Similarly, WGCNA analysis in Arabidopsis identified gene modules associated with cold stress and further revealed a key regulatory role for the transcription factor BPC6 in the plant’s response to various abiotic stresses [21,22].Although some progress has been made, the complete gene interaction network and variety-specific regulatory pathways underlying the rice cold stress response remain poorly defined. Thus, integrating transcriptomic data across multiple time points and varieties is urgently needed to construct a global regulatory map of this response and to elucidate the molecular basis of differential cold tolerance among rice varieties.

In this study, two rice varieties, DHX2 and QJ10, were used. Physiological and biochemical parameters were determined, and RNA sequencing was performed after low-temperature treatment at the booting stage. Unlike most previous transcriptomic studies on rice cold stress, which have focused primarily on the seedling stage, the present study focused on the booting stage—a stage that has a greater impact on yield yet has received much less research attention. Principal component analysis (PCA), cluster analysis, differential expression analysis, enrichment analysis, and transcription factor (TF) analysis were performed on the sequencing data. Furthermore, the physiological and biochemical indicators were integrated with weighted gene co-expression network analysis (WGCNA) to identify core modules associated with cold tolerance via module trait association analysis. Based on these results, several novel candidate regulatory factors were identified. These findings provide a theoretical foundation for a deeper understanding of the molecular mechanisms underlying rice tolerance to low temperatures during the booting stage, and offer new genetic resources for research on rice cold stress tolerance.

## 2. Materials and Methods

### 2.1. Plant Materials

Seeds of the cold-sensitive rice variety DHX2 and the cold-tolerant rice variety QJ10 were obtained from the Rice Research Institute of the Qiqihar Branch of the Heilongjiang Academy of Agricultural Sciences in China. Disinfect the seed surfaces with a 1% sodium chloride solution to remove contaminants. Soak the disinfected seeds in water at 37 °C for two days, then germinate them at 30 °C under a light cycle of 16 h (light)/8 h (dark). Plant the seeds in soil and place them in a greenhouse. Water every 5–7 days to maintain soil moisture at approximately 60%, and apply a compound fertilizer (N-P_2_O_5_-K_2_O = 20–20–20) at a concentration of 1 g/L monthly to promote growth and development. Plants were cultivated in controlled-environment greenhouses maintained at a temperature of 25 ± 3 °C and a relative humidity of 60–70%; lighting was provided exclusively by natural daylight, with no supplemental artificial illumination or shading applied. Rice plants grown to the booting stage were transferred to a cold chamber and subjected to cold stress treatment at 4 °C under a 12 h light/12 h dark cycle with a light intensity of approximately 200 μmol·m^−2^·s^−1^. Leaf samples of DHX2 and QJ10 were collected on days 0, 3, 5, and 7 of the cold stress treatment and stored at −80 °C. Three biological replicates were used for each sample.

### 2.2. Assays of Proline, POD, SOD, and MDA

A total of 0.1 g of the test sample was added along with 1 mL of extract, and subsequent enzyme activity assays were performed using the ice-bath homogenization method. The mixture was centrifuged at 4 °C and 12,000 rpm for 10 min, and the supernatant was collected.

#### 2.2.1. Measurement of MDA Content

MDA content was measured using the Malondialdehyde (MDA) Assay Kit (Beijing, China; catalog no. AKFA013M). The supernatant was incubated in a 95 °C water bath for 30 min, cooled on ice, and then centrifuged again at 25 °C and 12,000 rpm for 10 min. Finally, 200 μL of the resulting supernatant was collected, and the absorbance was measured at 532 nm and 600 nm, and the MDA content was calculated using the formula: MDA (μmol·L^−1^) = 6.45 × (A_532_ − A_600_) − 0.559 × A_450_ [23]. Each experiment was repeated three times.

#### 2.2.2. Measurement of POD Activity

POD activity was determined using the Peroxidase (POD) Assay Kit (Beijing, China; catalog no. AKAO005M). The supernatant was collected as the test solution. The absorbance value A_1_ was read immediately at 470 nm, and A_2_ was read 1 min later to calculate POD activity using the formula: POD (U·g^−1^·min^−1^) = 5000 × (A_2_ − A_1_) [24]. Each experiment was repeated three times.

#### 2.2.3. Measurement of SOD Activity

SOD activity was measured using the Superoxide Dismutase (SOD) Assay Kit (Beijing, China; catalog no. AKAO001M). The supernatant was placed in a 37 °C water bath for 30 min, and the absorbance was measured at 450 nm to calculate SOD activity using the formula: SOD (U·g^−1^) = 100 × (A_0_ − A)/A_0_ [25]. Each experiment was repeated three times.

#### 2.2.4. Measurement of Proline Content

Proline content was determined using the Proline Assay Kit (Beijing, China; catalog no. AKAM003M). A 200 μL aliquot of the supernatant was removed, the absorbance was measured at 520 nm, and the proline content was calculated using the formula: Proline (μg·g^−1^) = (A_520_ − b)/a × 50 [26]. Each experiment was repeated three times.

### 2.3. RNA-Seq and Differential Gene Expression Analysis

Total RNA was extracted from leaf samples (three replicates per time point) according to the manufacturer’s protocol. RNA-seq libraries were prepared using Illumina reagents and sequenced on the Illumina NovaSeq 6000 platform, generating 150 bp paired-end reads. Cleaned reads were processed using FastQC (v0.11.9) [27] for quality assessment and Trimmomatic (v0.39) [28] for removal of low-quality reads. Alignment was performed using HISAT2 (v2.2.1) [29] against the reference genome, and featureCounts (v2.01) [30] was used to count reads per gene. Genes with no mapped reads in any sample were removed. Differential expression analysis was conducted using DESeq2 [31], and significantly differentially expressed genes (DEGs) were identified based on an adjusted *p*-value < 0.05 and |log_2_FC| > 2. GO and KEGG enrichment analyses were performed using the clusterProfiler software (v4.14) [32].

### 2.4. WGCNA

Co-expression network analysis was performed using the WGCNA package in R [33]. In short, the FPKM matrix of all DEGs was used as input, and genes with low mean expression (FPKM < 2) were filtered out. The pickSoftThreshold function was used to determine the soft-thresholding power β. The smallest β value at which the signed scale-free topology fit R^2^ first reached ≥ 0.8 was selected for network construction. Subsequently, an adjacency matrix was constructed from the inter-gene correlation coefficients using the selected soft threshold, and a topological overlap matrix (TOM) was calculated. The TOM was converted to a dissimilarity matrix (1–TOM), and genes were clustered by average linkage hierarchical clustering. Modules were identified using the dynamic tree cut algorithm with a minimum module size of 30 genes, and similar modules were merged at a height threshold of 0.4. To associate co-expression modules with physiological indicators of cold stress (MDA, Pro, SOD, POD), we calculated the Pearson correlation coefficients and corresponding *p*-values between the module eigengene (ME) of each module and each indicator, and identified significantly correlated modules. For each module, we then calculated the correlation (kME) between each gene and the ME, and selected hub genes using the criterion |kME| > 0.80. Finally, the hub gene network was visualized using Cytoscape (v3.8.2) [34].

### 2.5. Construction of the Gene Regulatory Network (GRN)

Based on the annotations in PlantTFDB v5.0 (https://planttfdb.gao-lab.org/download.php accessed on 11 May 2026), 70 transcription factors were identified among the genes in the MEturquoise module, and 12 core transcription factors were selected by constructing a regulatory network using the STRING database [35]. Using machine learning, 356 genes that rapidly respond to cold stress were identified as potential regulatory factors. We employed the GENIE3 package in R [36] to calculate confidence levels (weights ≥ 0.25) and used the 424 inferred regulatory relationships as a candidate GRN. This network was visualized using Cytoscape (v3.8.2) [34].

### 2.6. qRT-PCR

Primer design (Supplementary Table S1) was performed using Primer Premier 5.0, and primer specificity was verified using the BLAST program in NCBI (https://ncbi.nlm.nih.gov accessed on 11 May 2026). Total RNA was extracted using an Ultra-Pure Total RNA Extraction Kit (Hangzhou Sumgen Biotech Co., Ltd., Hangzhou, China) and stored at −80 °C. First-strand cDNA (10 µL) was synthesized according to the instructions for the PrimeScript™ RT Master Mix (Takara Biomedical Technology (Beijing) Co., Ltd., Beijing, China). The internal reference gene was OsActin. qRT-PCR was performed in the LightCycler 96 system software using SYBR green (Vazyme Biotech Co., Ltd., Nanjing, China) and fluorescent dyes. Finally, the expression of the gene was calculated using the 2^−∆∆CT^ method [37].

## 3. Results

### 3.1. Physiological Response of Rice Under Cold Stress

**Figure 1:**
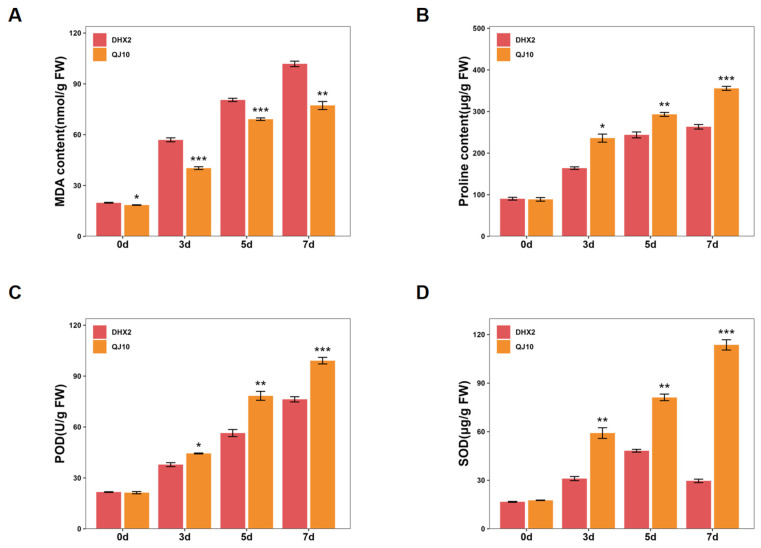
Physiological responses of DHX2 and QJ10 to cold stress at the booting stage. (**A**) MDA content. (**B**) Proline content. (**C**) POD content. (**D**) SOD content. Data are presented as mean ± SD (n = 3). Asterisks indicate significant differences between varieties (* *p* < 0.05, ** *p* < 0.01, *** *p* < 0.001).

To evaluate cold stress tolerance in the two varieties, DHX2 and QJ10 were subjected to 4 °C treatment for 7 days. Samples were collected at 0, 3, 5, and 7 days for measurement of superoxide dismutase (SOD), proline (Pro), peroxidase (POD), and malondialdehyde (MDA). As shown in Figure 1A–D, MDA levels in DHX2 were markedly higher than those in QJ10 over the 7-day cold stress period, whereas Pro, SOD, and POD levels were consistently lower. These findings indicate that QJ10 possesses greater cold stress tolerance than DHX2.

### 3.2. Analysis of Cold Stress-Induced Differentially Expressed Genes in Rice

To further investigate the molecular mechanisms underlying the differential cold stress responses of QJ10 and DHX2, we collected samples and performed RNA-seq analysis at 0, 3, 5, and 7 days post-treatment. Transcriptome sequencing of 24 root samples generated 158.4 Gb of clean reads. Each sample yielded over 6.60 Gb of clean data, with Q30 scores exceeding 90.7% and GC contents ranging from 50.92% to 53.60% (Supplementary Table S2), indicating that the RNA-seq data are of high quality. The PCA results showed that the samples formed tight within-group clusters and were clearly separated between groups. The first and second principal components accounted for 33.3% and 19.2% of the variance, respectively. This not only visually illustrated the differences among rice varieties under cold stress (Supplementary Figure S1), but also indicated that the experimental data were stable and reliable, and thus suitable for further analysis.

**Figure 2:**
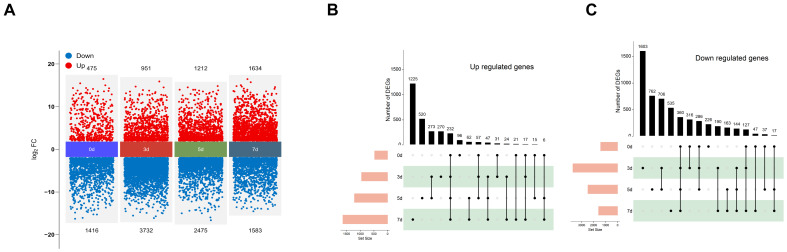
Transcriptomic profiles of QJ10 and DHX2 under cold stress at 0, 3, 5, and 7 days post-treatment. (**A**) Differentially expressed gene (DEG) counts in QJ10 relative to DHX2 at the indicated time points. (**B**) Upset plot of upregulated DEGs in QJ10 versus DHX2, showing unique and shared gene sets across time points. (**C**) Upset plot of downregulated DEGs in QJ10 versus DHX2. |log_2_FC| > 1, *p* < 0.05. Red, upregulation; blue, downregulation.

Differential expression analysis was performed with QJ10 as the treatment group and DHX2 as the control group (Figure 2A, Supplementary Table S3). Under a threshold of |log_2_(FC)| > 2 and *p*-value < 0.05, a total of 13,599 differentially expressed genes (DEGs) were identified. Overall, the total number of DEGs initially increased and then declined with prolonged cold stress; specifically, upregulated DEGs continued to accumulate, whereas downregulated DEGs peaked at 3 days of cold treatment. Overall, the total number of DEGs initially increased and then declined with prolonged cold stress; specifically, upregulated DEGs continued to accumulate, whereas downregulated DEGs peaked at 3 days of cold treatment. Further analysis revealed that the majority of upregulated DEGs occurred at 7 days of cold treatment, whereas downregulated DEGs were most abundant at 3 days, with 797 unique upregulated and 1487 unique downregulated genes identified (Figure 1B,C). Multi-point comparisons identified 232 DEGs consistently upregulated and 360 DEGs consistently downregulated across all four time points (Supplementary Table S4). GO and KEGG enrichment analyses (Supplementary Figure S2) revealed that upregulated genes were mainly associated with fundamental processes, including phospholipid metabolism, cellular organization, and glucose response, whereas downregulated genes were predominantly enriched in secondary metabolic pathways such as phenylpropanoid biosynthesis, lignin metabolism, and cytochrome P450-related functions.

### 3.3. Transcriptional Dynamics of Rice Under Cold Stress

**Figure 3:**
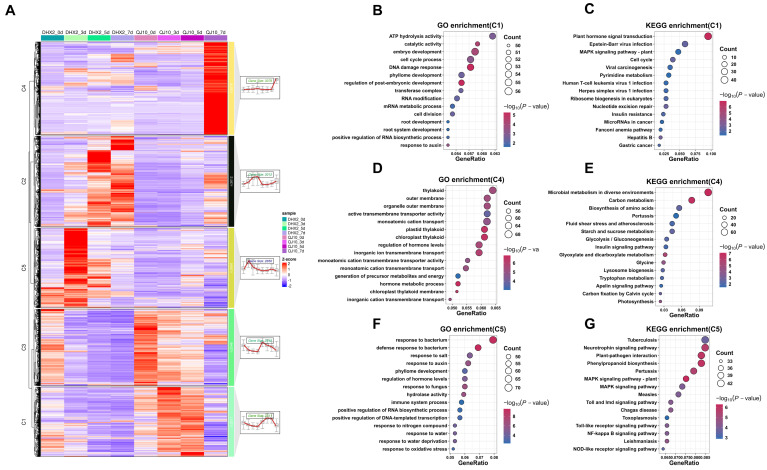
Differentially expressed gene clusters and functional annotations in QJ10 compared to DHX2. (**A**) Trend-based cluster analysis of gene expression in QJ10 and DHX2. (**B**) GO enrichment analysis of differentially expressed genes in Cluster 1. (**C**) KEGG enrichment analysis of differentially expressed genes in Cluster 1. (**D**) GO enrichment analysis of differentially expressed genes in Cluster 4. (**E**) KEGG enrichment analysis of differentially expressed genes in Cluster 4. (**F**) GO enrichment analysis of differentially expressed genes in Cluster 5. (**G**) KEGG enrichment analysis of differentially expressed genes in Cluster 5. Statistical significance was defined as *p*-value < 0.05.

To investigate the molecular basis of differential cold tolerance among varieties, we subjected the 13,599 DEGs to trend clustering analysis using Mfuzz. All genes were grouped into five clusters with distinct expression profiles (Figure 3A), revealing fundamental differences in the transcriptional reprogramming strategies of DHX2 and QJ10 under cold stress. Specifically, genes in Clusters 2 and 3 exhibited progressively elevated expression with prolonged cold treatment (0–7 days), whereas Cluster 4 genes displayed a marked upregulation in QJ10 at day 7, with no corresponding changes observed in DHX2. Additionally, Clusters 1, 4, and 5 displayed marked variety-specific expression: Clusters 1 and 4 were preferentially expressed in the cold-tolerant variety QJ10, whereas Cluster 5 was preferentially expressed in the cold-sensitive variety DHX2.

We next conducted GO and KEGG enrichment analyses for genes in Clusters 1, 4, and 5. The results revealed that genes in Clusters 1 and 4, which were preferentially expressed in QJ10, exhibited marked functional divergence from those in Cluster 5, which were preferentially expressed in DHX2 (Figure 3B–G). Specifically, genes in Cluster 1 were primarily enriched in growth- and development-related processes, including embryonic development, DNA damage response, cell cycle progression, and RNA modification; KEGG enrichment analysis further highlighted plant hormone signal transduction and cell cycle pathways. In contrast, genes in Cluster 4 were significantly enriched in components associated with ion transmembrane transport, hormone metabolism, and photosynthesis, with KEGG pathways predominantly involving carbon metabolism, photosynthesis, and amino acid biosynthesis. In marked contrast, genes in Cluster 5 were significantly enriched in defense responses to bacteria and fungi, salt and water stress responses, and immune system processes. Correspondingly, KEGG pathways were predominantly associated with defense and immunity, including plant–pathogen interaction, phenylpropanoid biosynthesis, MAPK signaling, and Toll/Imd signaling. Collectively, the two varieties exhibited distinctly divergent transcriptional reprogramming strategies under cold stress.

### 3.4. Analysis of Co-Expression Modules in Rice Under Cold Stress

**Figure 4:**
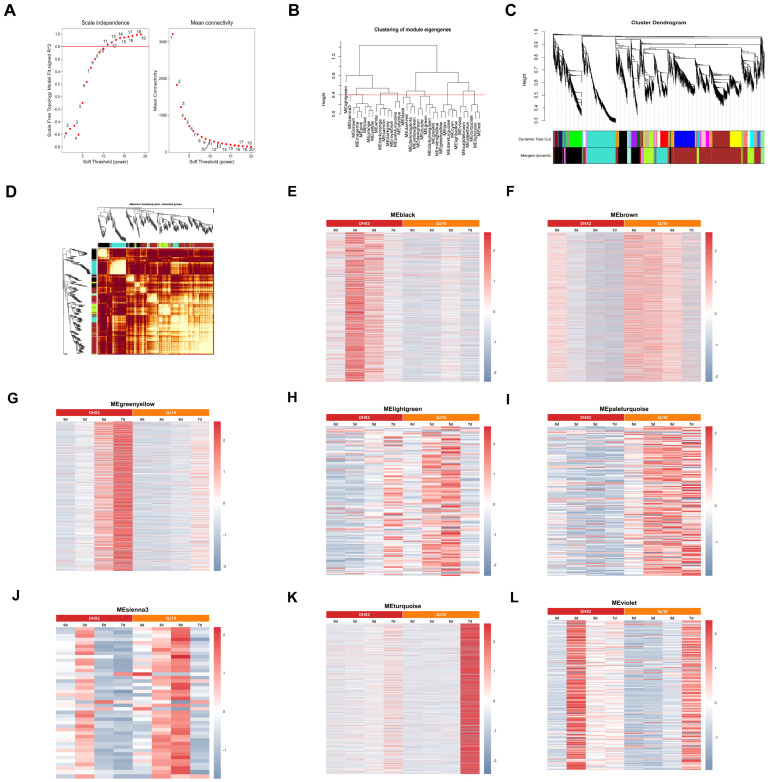
Weighted gene co-expression network analysis (WGCNA) of differentially expressed genes between QJ10 and DHX2 under cold stress. (**A**) Analysis of network topology for various soft-thresholding powers, showing scale independence and mean connectivity. (**B**) Cluster dendrogram of module eigengenes and module merging at a dissimilarity threshold of 0.4. (**C**) Hierarchical clustering tree of differentially expressed genes, with modules indicated by color bands and individual genes represented by branch tips. (**D**) Heatmap of correlations among module eigengenes. Color intensity reflects the strength of correlation, with darker yellow indicating higher correlation. Expression patterns of genes in eight co-expression modules (**E**) MEblack, (**F**) MEbrown, (**G**) MEgreenyellow, (**H**) MElightgreen, (**I**) MEpaleturquoise, (**J**) MEsienna3, (**K**) MEturquoise, and (**L**) MEviolet. Expression values were normalized by Z-score transformation, and relative expression levels are represented by the color scale.

To identify key regulatory factors involved in the rice cold stress response, we filtered out low-expression genes from the initial 13,599 DEGs, retaining 9270 DEGs for subsequent WGCNA. We performed network topology analysis with soft-thresholding powers (β) ranging from 1 to 40, and identified a power value at which scale independence and mean connectivity were relatively balanced (Figure 4A). We selected the minimum β at which R^2^ first exceeded 0.8, ultimately determining β = 11 (0.81) (Figure 4A). Subsequently, a hierarchical cluster-based gene tree was constructed, yielding 11 co-expression modules. After merging similar modules at a height threshold of 0.4, a total of 8 modules were obtained. All genes within the same module shared similar expression patterns, and the number of DEGs per module ranged from 43 (MEsienna3) to 3931 (MEbrown) (Figure 4B,C; Supplementary Table S5). The similarity among modules was evaluated by correlating their module eigengenes (Figure 4D).

Analysis of module eigengene expression patterns revealed that genes in five modules, namely MEbrown, MElightgreen, MEpaleturquoise, MEsienna3, and MEturquoise, exhibited significantly higher expression levels in QJ10 than in DHX2 across all three cold stress time points. Moreover, the expression of DEGs within these modules was elevated at all cold stress stages relative to 0 days (Figure 4F,H–K). Genes in the MEblack and MEgreenyellow modules exhibited lower expression levels in QJ10 than in DHX2 throughout cold stress. For the MEviolet module, expression in QJ10 was lower than in DHX2 at day 3, but higher at day 7 (Figure 4E,G,L). In summary, the co-expression modules of QJ10 and DHX2 under cold stress displayed pronounced cultivar specificity. QJ10 exhibited sustained high expression or delayed upregulation across multiple modules, whereas DHX2 showed generally lower expression or transient fluctuations.

### 3.5. Identification of Gene Modules Associated with Cold Stress Responses in Rice

**Figure 5:**
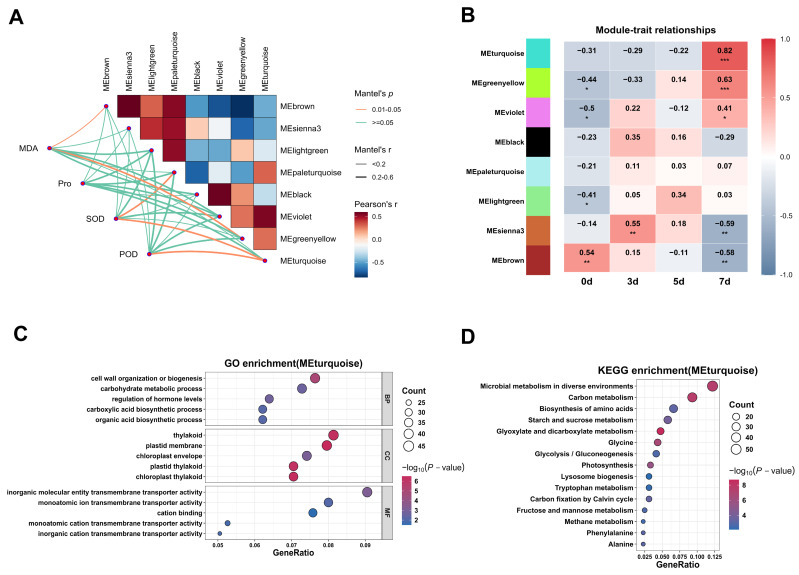
Module–trait association analysis and identification of core modules based on WGCNA. (**A**) Heatmap of correlations between module eigengenes (ME) and cold-stress physiological indicators. (**B**) Correlation between each module eigengene and the duration of cold stress treatment. (**C**) GO enrichment analysis of genes in the MEpaleturquoise module. (**D**) KEGG enrichment analysis of genes in the MEpaleturquoise module. Color intensity reflects the magnitude of the correlation coefficient; * *p* < 0.05, ** *p* < 0.01, *** *p* < 0.001.

Based on the WGCNA results, we evaluated the module–trait associations by calculating the Pearson correlation coefficients between module eigengenes (ME) and physiological traits (Figure 5A, Supplementary Table S6). The results showed that MEbrown had a significant negative correlation with MDA, whereas MEgreenyellow had a positive correlation. MEpaleturquoise was positively correlated with SOD, and MEturquoise showed positive correlations with both SOD and POD. We further calculated the Pearson correlations between module eigengenes (ME) and the duration of cold stress treatment, and identified one module significantly associated with treatment duration (|kME| > 0.80, *p* < 0.05) (Figure 5B). MEturquoise was positively correlated with treatment duration (|kME| = 0.82, *p* < 0.05). GO enrichment analysis of the MEturquoise module genes (Figure 5C) showed that, in biological processes, these genes were significantly enriched in terms related to cell wall organization or biogenesis, carbohydrate metabolic process, regulation of hormone levels, organic acid biosynthetic process, and carboxylic acid biosynthetic process. In the cellular component category, they were mainly localized to thylakoids, plastid membranes, chloroplast envelopes, chloroplast thylakoids, and plastid thylakoids. In the molecular function category, they were primarily involved in inorganic molecular entity transmembrane transporter activity, monoatomic ion transmembrane transporter activity, cation binding, and monoatomic cation transmembrane transporter activity. KEGG pathway enrichment analysis (Figure 5D) revealed that genes in the MEturquoise module were highly enriched in pathways related to microbial metabolism in diverse environments, carbon metabolism, biosynthesis of amino acids, starch and sucrose metabolism, glyoxylate and dicarboxylate metabolism, glycine metabolism, glycolysis/gluconeogenesis, photosynthesis, carbon fixation by the Calvin cycle, and tryptophan metabolism.

### 3.6. Identification of Core Transcription Factors in the Cold Stress Response

**Figure 6:**
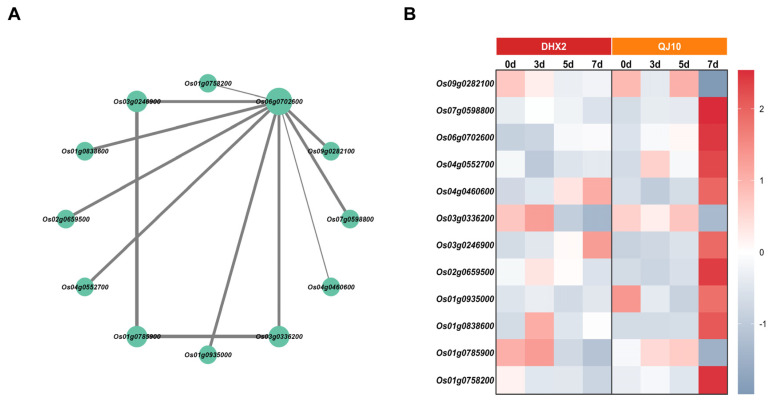
Hub transcription factors responsive to cold stress in the MEturquoise module and their co-expression network. (**A**) Co-expression network of the 12 hub transcription factors. Gray lines indicate correlations between hub transcription factors; node size represents the |kME| value, and line thickness indicates co-expression strength. (**B**) Heatmap showing the expression of the 12 hub transcription factors in QJ10 and DHX2. The values represent Z-score-normalized FPKM across four time points under cold stress treatment; red indicates high expression, and blue indicates low expression.

Transcription factors (TFs) are key regulators of plant development and cold stress responses. To identify key transcription factors involved in the cold stress response, we selected 12 TFs from the MEturquoise module. These TFs belong to families such as Dof, C2H2 zinc finger, LBD (lateral organ boundary domain), bZIP, ARF (auxin response factor), and NAC. They form a regulatory network that may play a key role in reducing cold tolerance in rice (Figure 6A). We analyzed the expression patterns of these 12 TFs in QJ10 and DHX2 under cold stress and found that nine of them were strongly induced in QJ10 at 7 days of treatment, with expression levels significantly higher than those in DHX2. For example, *Os01g0758200* (*OsDof6*), *Os01g0838600* (*C2H2*), *Os01g0935000* (*C2H2*), *Os02g0659500* (*C2H2*), *Os03g0246900* (*LBD*), *Os04g0460600* (*OsNAC2*), *Os04g0552700* (*C2H2*), *Os06g0702600* (*OsARF19*), and *Os07g0598800* (*C2H2*) were upregulated at 7 days of cold stress treatment in QJ10, whereas the same genes in DHX2 were mostly downregulated under the same treatment. The other two TFs, *Os01g0785900* (*C2H2*) and *Os03g0336200* (*OsbZIP30*), showed low expression in both varieties at the late stage of cold stress (7 days). In contrast, *Os09g0282100* (*C2H2*) remained downregulated in DHX2, while in QJ10 it displayed a fluctuating pattern, with upregulation at 5 days and downregulation at 7 days. Overall, the expression levels of these nine TFs, which were strongly induced during the late stages of cold stress in QJ10, were higher in QJ10 than in DHX2, suggesting that these TFs may be key regulators of cold tolerance in QJ10 (Figure 6B).

### 3.7. Inference of Transcription Factor Regulatory Networks Using Machine Learning

WGCNA effectively identifies gene co-expression networks and hub genes but provides limited insight into direct regulatory relationships among genes. Thus, although the 12 transcription factors identified above represent reliable candidates for the cold stress response network (Figure 6A), the regulatory relationships among them remain unresolved. To clarify these relationships, we applied the machine learning-based GENIE3 package to predict regulatory interactions between genes and transcription factors.

**Figure 7:**
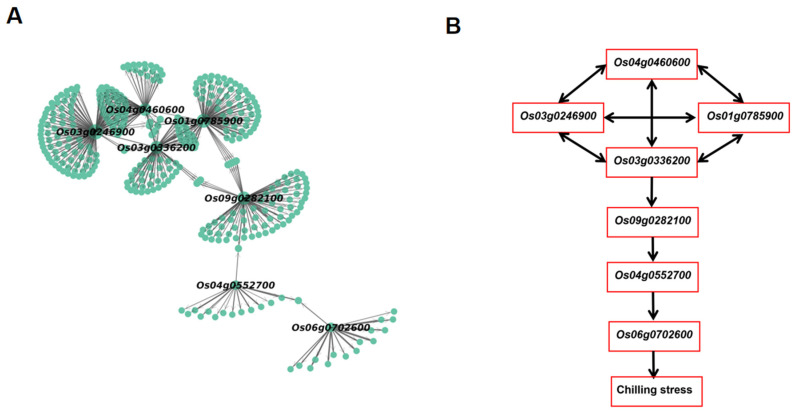
GENIE3-inferred regulatory network of core transcription factors associated with the cold stress response. (**A**) Transcriptional regulatory network centered on seven core transcription factors. Each node represents a gene, with lines denoting regulatory relationships and gray arrows indicating the direction of regulation. Circular nodes correspond to non-TF genes. (**B**) Mutual regulatory interactions among the seven core transcription factors.

We used GENIE3, a random forest-based algorithm, to predict potential regulatory relationships among 356 cold-stress-responsive genes (Supplementary Table S7). By further mapping these genes against the 12 previously identified hub transcription factors, we identified seven key TFs potentially involved in the cold stress regulatory network: *Os01g0785900* (*C2H2*), *Os03g0336200* (*OsbZIP30*), *Os06g0702600* (*OsARF19*), *Os09g0282100* (*C2H2*), *Os04g0552700* (*C2H2*), *Os03g0246900* (*LBD*), and *Os04g0460600* (*OsNAC2*). The predicted regulatory network formed by these transcription factors indicates that *OsNAC2* may be located downstream in this network (Figure 7A). *OsNAC2* is hypothesized to integrate cold stress signals and regulate the expression of downstream genes (Figure 7B). However, these regulatory relationships require further validation.

### 3.8. Validation of Candidate Transcription Factor Expression Patterns Under Cold Stress

**Figure 8:**
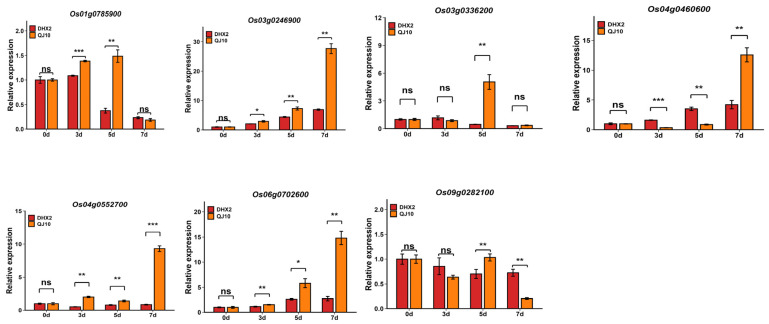
qRT-PCR validation of seven candidate transcription factors in leaves of DHX2 and QJ10 under cold stress. Data are shown as mean ± SD (n = 3 biological replicates). Relative expression levels were calculated using the 2^−ΔΔCT^ method with OsActin as the internal control. Asterisks indicate significant differences between QJ10 and DHX2 (ns *p* > 0.05, * *p* < 0.05, ** *p* < 0.01, *** *p* < 0.001; Student’s *t*-test). Red and orange represent the cold-sensitive variety DHX2 and the cold-tolerant variety QJ10, respectively.

To further validate the expression patterns of the seven candidate transcription factors in DHX2 and QJ11, we used qRT-PCR to analyze their relative expression levels in the leaves of both varieties at 0, 3, 5, and 7 days after cold stress treatment. In the cold-resistant variety QJ10, most of the candidate transcription factors were significantly induced by cold stress (Figure 8). The expression level of *Os03g0246900* (*LBD*) increased steadily with the duration of cold treatment, reaching approximately 29.3-fold relative to the control at 7 days. *Os06g0702600* (*ARF19*) and *Os04g0460600* (*OsNAC2*) also showed significant time-dependent increases; at 7 days, their expression levels were 16.1-fold and 13.8-fold higher than those of the control, respectively. *Os04g0552700* (*C2H2*) showed a significant increase at 3 days and reached 9.7-fold of the control level at 7 days. *Os01g0785900* (*C2H2*) and *Os03g0336200* (*bZIP30*) exhibited an initial increase followed by a decline: they were significantly induced at 3 and 5 days, and returned to the basal level at 7 days. *Os09g0282100* (*C2H2*) showed little overall change in expression, with a statistically significant but limited difference observed only at 5 days. 

The cold response of genes in the cold-sensitive variety DHX2 was significantly weaker than that in QJ10, with most genes showing reduced upregulation and a slower response.

## 4. Discussion

Under cold stress, plants primarily respond by enhancing membrane integrity, antioxidant capacity, and osmotic regulation [38]. As a product of membrane lipid peroxidation, the level of MDA under stress conditions reflects the degree of plant resistance [39]. Comparison of the cold-tolerant variety QJ10 and the cold-sensitive variety DHX2 revealed that MDA content in QJ10 was significantly lower than that in DHX2, suggesting reduced membrane lipid peroxidation and better preservation of membrane integrity under cold stress (Figure 1A). Proline, an essential osmoregulator, plays a critical role in maintaining cellular osmotic balance and promoting water uptake to mitigate dehydration [40]. The significantly higher proline content in QJ10 relative to DHX2 (Figure 1B) indicates enhanced osmotic regulation capacity in this variety. The SOD and POD antioxidant systems effectively scavenge reactive oxygen species (ROS) in plants, mitigating oxidative damage [41]. The SOD and POD activities in QJ10 were significantly higher than those in DHX2 (Figure 1C,D), reflecting enhanced reactive oxygen species scavenging capacity and consequently reduced oxidative damage.

RNA sequencing (RNA-seq) is a rapid, cost-effective, and high-throughput research method [42]. RNA-seq analysis has enabled the identification and characterization of numerous stress-responsive genes, providing critical data for an in-depth understanding of the molecular regulatory networks underlying plant adaptation to adverse conditions [43,44,45]. In this study, transcriptomic sequencing analysis of QJ10 and DHX2 under cold stress revealed that the number of differentially expressed genes in QJ10 relative to DHX2 initially increased and then declined. The number of upregulated genes continued to rise, whereas the number of downregulated genes peaked at 3 days. Persistently upregulated genes were enriched in basic metabolic processes, while persistently downregulated genes were associated with secondary metabolic pathways (Supplementary Figure S2). This suggests that QJ10 may exhibit greater cold tolerance by suppressing secondary metabolism to conserve energy while simultaneously enhancing the adaptability of its basal metabolism. Trend clustering analysis represents an effective method for identifying biologically meaningful dynamic expression patterns among DEGs [46]. In this study, trend clustering analysis of differentially expressed genes revealed that Clusters 1, 4, and 5 exhibited similar expression trends between the two varieties. Genes in Clusters 1 and 4 were predominantly expressed in QJ10, whereas those in Cluster 5 were predominantly expressed in DHX2 (Figure 3A). This observation suggests that the differences in cold stress tolerance between QJ10 and DHX2 may be attributable to the differential expression of these three gene clusters. Previous studies have shown that under cold stress, the cold-tolerant tobacco variety DFH strongly induces the upregulation of numerous genes, with marked enrichment of the oxidative phosphorylation pathway. This enrichment provides the energy required for antioxidant synthesis and the accumulation of soluble sugars and proline, thereby enhancing membrane stability and osmotic regulation. In contrast, the cold-sensitive variety BJYD lacks such metabolic activation, which constitutes an important molecular basis for its heightened sensitivity to cold [47]. In contrast, the genes in Clusters 1 and 4 that were upregulated in QJ10 in this study are primarily involved in growth-regulating pathways such as the cell cycle, DNA repair, and hormone signaling, suggesting that QJ10 resists cold stress by maintaining growth homeostasis. Conversely, the fifth cluster of genes preferentially expressed in DHX2 is concentrated in defense and immune-related pathways, reflecting that DHX2 redirects more metabolic resources toward stress responses rather than growth maintenance (Figure 3B–F).

Transcription factors (TFs) play a key role in regulating plant growth and development and in responses to abiotic stress [48]. WGCNA is an effective method for exploring gene co-expression networks and identifying hub genes therein [49]. In this study, the MEturquoise module was identified as a core module that showed a significant positive correlation with both the duration of cold stress treatment and the activities of antioxidant enzymes (SOD and POD) (Figure 5A). This module was significantly enriched in processes such as carbohydrate metabolism, starch and sucrose metabolism, glycolysis/gluconeogenesis, and organic acid biosynthesis, with a large number of genes localized to the chloroplast thylakoid membranes and plastid membranes. At the molecular function level, it is primarily involved in cation binding and the transmembrane transport of inorganic ions. These results indicate that the MEturquoise module is significantly enriched in pathways related to carbohydrate metabolism and ion transport, suggesting that this module may support the antioxidant system by regulating these processes. Further analysis of the genes within this module identified 12 key transcription factors associated with the cold stress response in rice; most of these transcription factors belong to gene families known to be involved in abiotic stress responses (Figure 6). C2H2-type zinc finger proteins play a key role in the plant response to abiotic stress. In rice, *OsC2H2.35* negatively regulates the expression of cold-responsive genes by binding to the promoters of *OsDREB1A* and *OsDREB1C* [50]. *OsNAC2* is induced by low temperature, drought, and salt stress; its overexpression significantly enhances rice tolerance to cold, salt, and drought and improves cell membrane stability [51]. As an auxin-responsive factor, *OsARF19* acts in concert with *ARF7* to activate CRF3 expression under cold stress through degradation of the Aux/IAA repressor protein, thereby reshaping root architecture in response to low temperatures [52]. *PtrLBD41*, a poplar homolog of the *LBD* family transcription factor *Os03g0246900*, responds to various abiotic stresses, including low temperature, dehydration, and ABA [53]. *OsDof1*, a homolog of *OsDof6*, enhances rice cold tolerance by upregulating the expression of several cold-responsive genes, including *OsDREB1B* and *OsLEA3* [54]. Therefore, the above data suggest that these 12 transcription factors are potential regulators of the rice cold stress response, although their specific functions under low temperature conditions require further validation.

Although WGCNA is effective at identifying hub genes within co-expression clusters, directly assessing the regulatory relationships between different genes remains difficult [55]. To address this limitation, we employed the machine learning algorithm GENIE3, which infers regulatory networks from a series of regression trees [56]. This study further employed the GENIE3 algorithm, a random-forest-based approach, to predict and construct a cold stress response regulatory network centered on seven transcription factors, including *OsNAC2*. Notably, *OsNAC2* emerged as a key regulatory node within this predicted network. This is highly consistent with previous studies, as extensive evidence has shown that *OsNAC2* is a key positive regulator of rice cold tolerance: it is strongly induced by low temperatures and exerts its effects through both *CBF*-dependent and *CBF*-independent pathways [57]. The central role of *OsNAC2* in the predicted network not only validates the reliability of the analytical strategy employed in this study but also suggests that the remaining six co-regulated transcription factors (such as *OsARF19* and members of the *LBD* family) may participate in the cold stress response through synergistic or hierarchical interactions with *OsNAC2*. The identification of these key transcription factors involved in the cold stress response provides a reliable strategy and new entry points for elucidating the complex transcriptional regulatory hierarchy underlying the rice response to cold stress.

## 5. Conclusions

Through physiological, transcriptomic, and WGCNA analyses, this study revealed the differences between the cold-tolerant variety QJ10 and the cold-sensitive variety DHX2 under cold stress. QJ10 exhibited lower MDA content and higher levels of Pro, SOD, and POD. Transcriptomic analysis revealed that QJ10 primarily maintained the expression of genes associated with growth, development, and basal metabolism, whereas DHX2 tended to activate defense responses. The MEturquoise module, identified through WGCNA, showed significant positive correlations with SOD and POD activities and the duration of cold treatment, and was enriched in pathways such as carbon metabolism, photosynthesis, and ion transport. Twelve hub transcription factors were identified from this module, nine of which showed elevated expression at the late stages of cold stress in QJ10. Further analysis combining GENIE3 predictions and qRT-PCR validation confirmed that seven transcription factors, including OsNAC2, OsLBD, and OsARF19, were significantly induced by cold stress in QJ10. The results described above provide candidate regulatory factors and co-expression networks associated with cold tolerance in rice. However, the predicted regulatory relationships still require functional validation, thus laying the groundwork for a deeper understanding of the molecular mechanisms underlying cold tolerance.

## Data Availability

The original contributions presented in the study are included in the article; further inquiries can be directed to the corresponding authors.

## Supplementary Materials

The following supporting information can be downloaded at: https://www.mdpi.com/article/10.3390/genes17060639/s1. Figure S1: RNA-Seq PCA; Figure S2: GO and KEGG enrichment analysis of differentially expressed genes across four time points of cold stress; Table S1: Gene RT-qPCR primers; Table S2: The quality of the transcriptome sequencing data was assessed; Table S3: Analysis of expression differences under cold stress conditions; Table S4: DEGs that were up-regulated and down-regulated at all four time points during cold stress; Table S5: Summary of genes in each WGCNA module; Table S6: Correlations between Module Eigengenes and Physiological Indicators; Table S7: GENIE3 regulatory network predictions and weight matrices for 356 cold-stress-responsive genes.
