# Supplementary figures and images for: Transcriptomic and Weighted Gene Co-Expression Network Analysis Reveals Molecular Regulatory Mechanisms of Cold Stress in Rice
Source: Genes (Basel). 2026 May 31;17(6):639. doi: 10.3390/genes17060639 (PMC13298825; doi:10.3390/genes17060639)

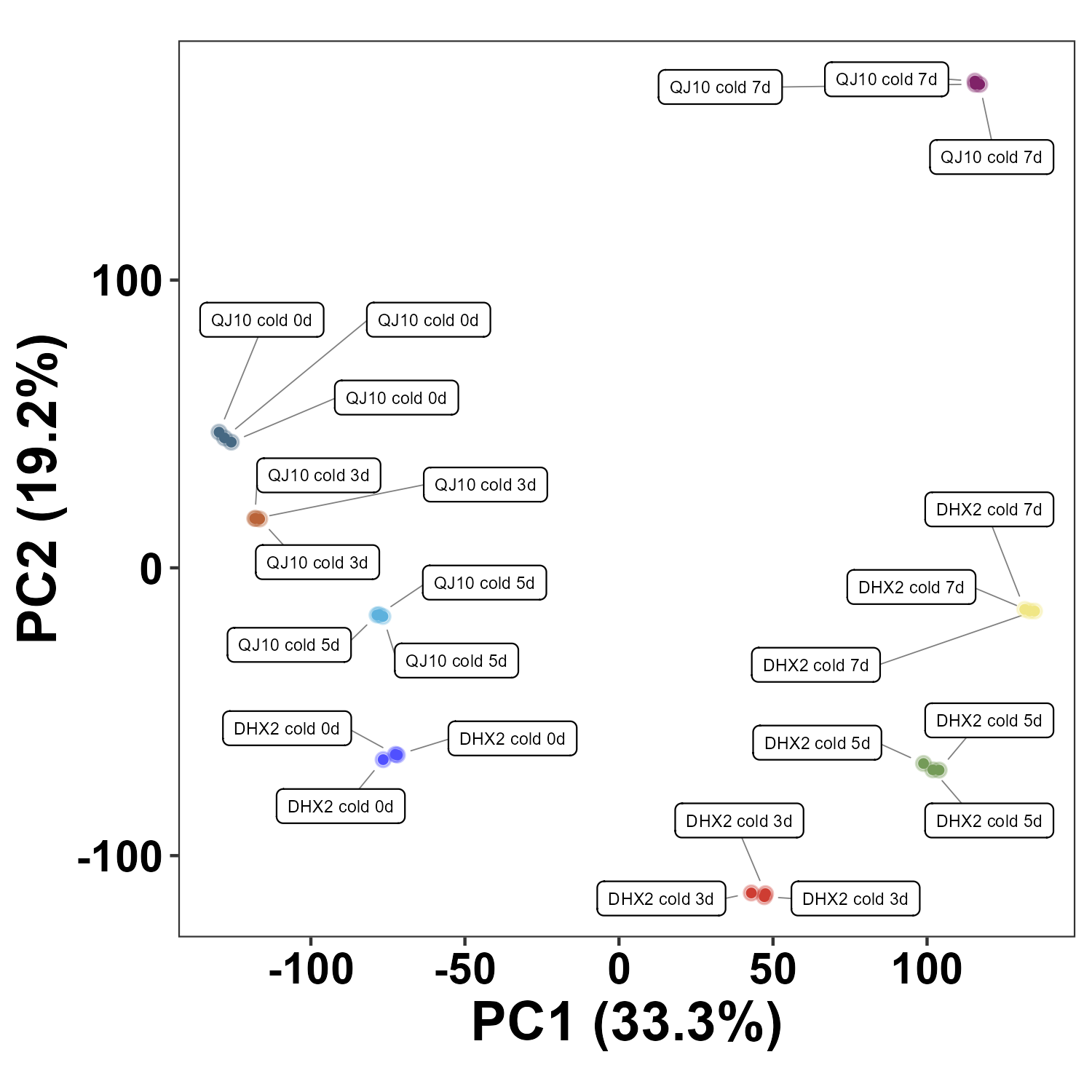

Supplement: Supplementary file 1 [file genes-17-00639-s001.zip › Supplementary Figure S1.tif]

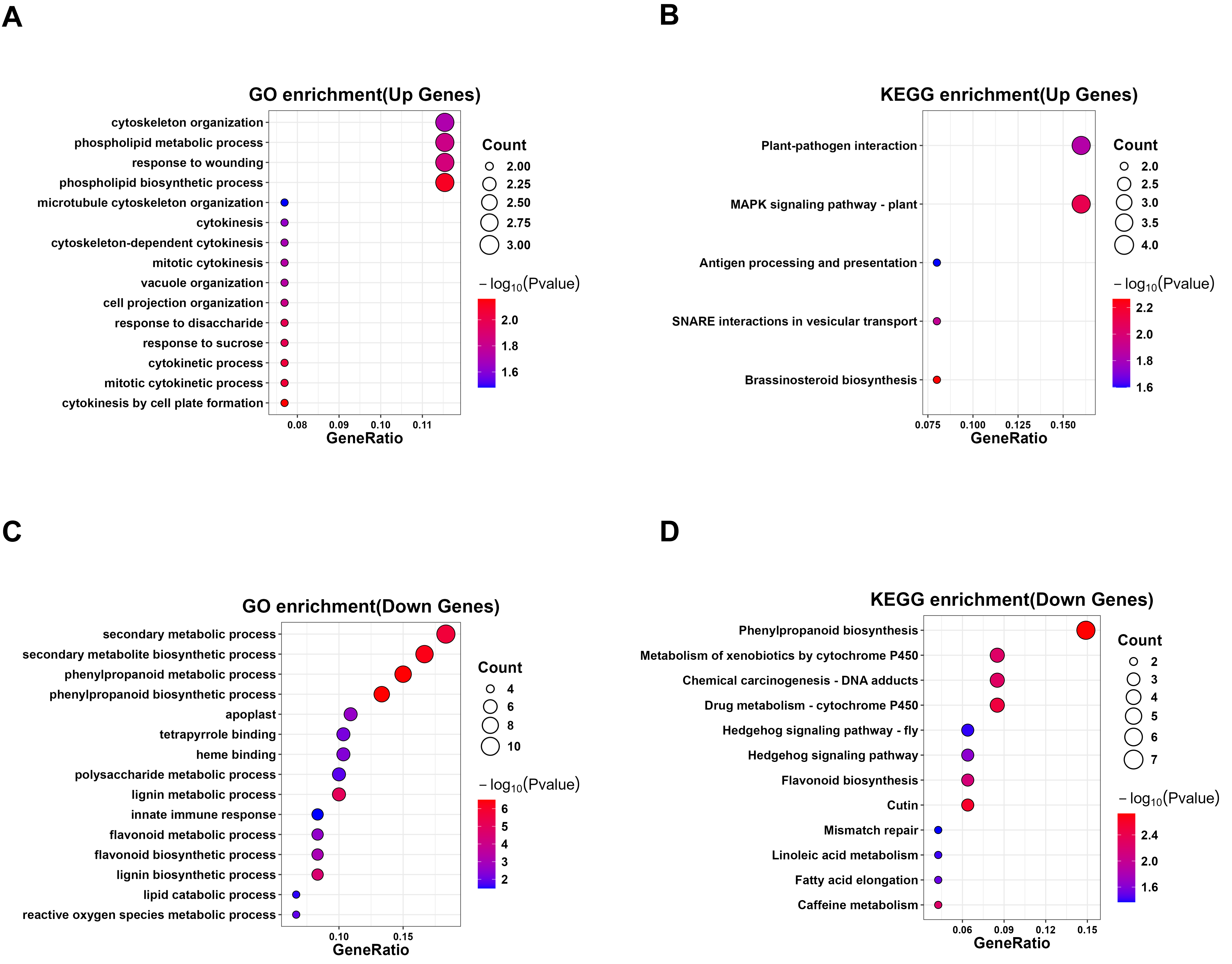

Supplement: Supplementary file 1 [file genes-17-00639-s001.zip › Supplementary Figure S2.tif]
